# Supplementary material for: Mechanisms governing the pioneering and redistribution capabilities of the non-classical pioneer PU.1
Source: Nat Commun. 2020 Jan 21;11:402. doi: 10.1038/s41467-019-13960-2 (PMC6972792; doi:10.1038/s41467-019-13960-2)
Supplement: Supplementary file 7 — Source data [file 41467_2019_13960_MOESM7_ESM.zip › Source_Data/FigureS2/FigureS2B_MotifScanOutput/KmeansCluster14/homerResults/motif7.similar.html]

motif7

## Information for motif7

A
C
T
G
C
T
A
G
G
T
A
C
T
C
A
G
C
A
T
G
C
A
T
G
T
G
C
A
G
T
A
C
C
G
A
T
C
G
T
A
G
T
C
A
  
Reverse Opposite:  

C
A
G
T
G
C
A
T
G
C
T
A
C
A
T
G
A
C
G
T
G
T
A
C
G
T
A
C
A
G
T
C
C
A
T
G
G
A
T
C
T
G
A
C
  

|  |  |
| --- | --- |
| p-value: | 1e-22 |
| log p-value: | -5.074e+01 |
| Information Content per bp: | 1.618 |
| Number of Target Sequences with motif | 86.0 |
| Percentage of Target Sequences with motif | 16.86% |
| Number of Background Sequences with motif | 2234.7 |
| Percentage of Background Sequences with motif | 4.93% |
| Average Position of motif in Targets | 99.8 +/- 51.9bp |
| Average Position of motif in Background | 94.9 +/- 70.2bp |
| Strand Bias (log2 ratio + to - strand density) | -0.5 |
| Multiplicity (# of sites on avg that occur together) | 1.15 |
| Motif File: | file (matrix) reverse opposite |

### Similar de novo motifs found

|  |  |  |  |  |  |  |  |
| --- | --- | --- | --- | --- | --- | --- | --- |
| Rank | Match Score | Redundant Motif | P-value | log P-value | % of Targets | % of Background | Motif file |
| 1 | 0.808 | C T G A C T A G C T A G A C T G C T A G G T A C T C A G A C T G C T A G T G C A A G T C G A C T G T C A | 1e-20 | -47.753659 | 14.71% | 4.02% | motif file (matrix) |
| 2 | 0.897 | A C T G A C T G G A T C A C T G A C T G A C T G C T G A G T A C A C G T C G T A C T G A C T A G | 1e-18 | -42.616294 | 6.67% | 0.85% | motif file (matrix) |
